# Role of Serological Tests in the Diagnosis of Mold Infections

**DOI:** 10.1007/s12281-018-0321-1

**Published:** 2018-09-05

**Authors:** Malcolm Richardson, Iain Page

**Affiliations:** 1 Mycology Reference Centre, ECMM Centre of Excellence in Clinical and Laboratory Mycology and Clinical Studies, Wythenshawe Hospital, Manchester University NHS Foundation Trust, Manchester, M23 9LT UK; 2 Division of Infection, Inflammation and Respiratory Medicine, Faculty of Biology, Medcine and Health, The University of Manchester, Manchester, M13 9PL UK; 3 National Aspergillus Centre, Manchester University NHS Foundation Trust, Wythenshawe Hospital, Manchester, M13 9PL UK

**Keywords:** Molds, Serology, Antibody assays, Filamentous fungi, Aspergillus, Talaromyces, Chronic pulmonary aspergillosis, Allergic bronchopulmonary aspergillosis, Allergic bronchopulmonary mycoses, Mucormycosis, Pythiosis

## Abstract

**Purpose of Review:**

To understand the role of antibody detection in the diagnosis of infections caused by filamentous fungi (molds). Rapid and accurate profiling of infection-causing fungal pathogens remains a significant challenge in modern health care. Classical fungal culture and serology continue to be relevant even though over the past few decades, antigen (biomarker) assays such as ELISA and lateral flow devices have been developed and validated.

**Recent Findings:**

This article reviews the current antibody detection systems (serological tests) for the diagnosis of mold infections associated with pulmonary disease and introduces new developments. Classic and more recently developed serological techniques and their performance characteristics, including immunodiffusion, complement fixation, and ELISA.

**Summary:**

The diseases covered are allergic bronchopulmonary aspergillosis, chronic pulmonary aspergillosis, invasive aspergillosis, mucormycosis, diseases caused by filamentous basidiomycetes, infection caused by *Talaromyces marneffei* and pythiosis. Serology remains a cornerstone for fungal diagnostic testing.

## Introduction

This review will focus on the role of serology in the diagnosis of fungal infections caused by filamentous fungi. In practice, the term serology used in this context refers to the diagnostic identification of antibodies against these pathogens in serum. In 2013, Mark Lindsay wrote in this journal: “Finally, the future of fungal serology is still to be seen. While future molecular technologies may supplant serologic methodologies, molecular methods for performance directly from patient specimens still need standardization including improvement in time, technology and cost. The current complexity of fungal serology testing relegates most antibody testing and some antigen testing to only larger clinical and reference laboratories. For fungal serology to continue to progress, development of sufficiently sensitive and specific, rapid and simple methods that can be performed at local laboratories is necessary for improved turn-around-times and earlier initiation of specific therapy” [1]. The present review will examine whether the sentiments expressed above have been fulfilled with respect to mold (filamentous, and filamentous-like fungi) and will highlight new developments in this area.

Serologic tests for the detection of antibodies have been useful for non-culture-based diagnosis of fungal infection since the 1950s. Available technologies include immunodiffusion (ID), complement fixation (CF), and enzyme immunoassay (EIA). Lateral flow devices for specific antibodies are being developed and validated.

There are many advantages in using serology for diagnosis of invasive mold infection. Firstly, results may be positive when culture results are negative or samples are difficult to obtain. In the Mycology Reference Centre Manchester, approximately 50% of sputum samples from patients with chronic forms of aspergillosis are negative in culture. Secondly, if positive, serological results may reduce the need for culture. Finally, serology is a minimally invasive sample, which lowers hindrances to testing. Well-recognized and documented disadvantages of serology include low levels of sensitivity and specificity. A negative serological test should not exclude the presence of fungal infection. Some tests, particularly CF, are time-consuming and technically demanding. Patients who are immunocompromised may have a reduced antibody response, which would dramatically reduce the value of serological assays [2]. Interpretation of serological results may be confounded by the inability of serology that measures specific immunoglobulins to distinguish between current or previous infection. False positives may occur with some tests. Finally, sensitivity is dependent on the type of disease and the timing of testing relative to the disease process, for example, early infection compared with late.

Serology is useful for the diagnosis of some invasive mycoses, particularly when used in association with microscopical and culture procedures. Serological assays involve both qualitative and quantitative assessment of the circulating antibodies in blood. Depending on the method employed, semi-quantitative and quantitative, serological assays may have potential prognostic value, although they do pose some problems in terms of specificity and/or sensitivity (reviewed in [3–5]). In addition, some kits are not currently commercially available, and there is a lack of standardization. Among the common methods used, ID shows poor sensitivity, but if conducted with reference infection-specific antigens and antisera, it has high specificity.

## Aspergillosis

A small number of *Aspergillus* species, most commonly *Aspergillus fumigatus*, may lead to a variety of allergic states and chronic disease and, rarely, life-threatening invasive aspergillosis. This occurs primarily in patients with severe immunodeficiency and has dramatically increased in recent years. Measuring *Aspergillus*-specific antibodies is an important part of the diagnostic pathway for allergic bronchopulmonary aspergillosis (ABPA) and chronic pulmonary aspergillosis (CPA) (reviewed exhaustively in [3, 4]). These infections are likely to represent a major public health issue on a global scale as 20–35% of patients develop *Aspergillus*-specific antibodies following tuberculosis treatment, and 63% of these develop pulmonary aspergillosis within 3 years (reviewed in [3]). The global 5-year period prevalence of (CPA) secondary to tuberculosis has been estimated to be between 0.8 and 1.37 million cases. As *Aspergillus fumigatus* is the primary causative agent, isolation of the responsible *Aspergillus* species from the airway tract has a role in diagnosis and is essential to identify drug resistance, but unfortunately, the rate of isolation from sputum is relatively low with current standard culture techniques [5].

A number of recent reviews have examined the utility of antibody detection in the diagnostic work-up of chronic manifestations of aspergillosis [3, 4]. Here, we appraise recent developments and new approaches.

### Chronic Pulmonary Aspergillosis

CPA is possibly the fungal infection where serology is most useful. It affects more people globally than any other serious fungal infection, most commonly as a complication of pulmonary tuberculosis, although other chronic lung diseases such as emphysema and sarcoidosis can also be complicated by CPA.

While serum antigen tests such as galactomannan are the key to diagnosing acute invasive aspergillosis, they are only positive in around 25% of CPA patients [6, 7]. Antibody tests are, however, positive in over 90% of patients [8]. These two facts may be linked, as galactomannan is one of the immune-dominant antigens against which IgG antibodies are formed. A recently developed commercial anti-galactomannan IgG assay (Dynamiker, China) has a sensitivity of 77% in CPA cases [9]. Although these figures come from different clinical cohorts, it appears that similar numbers of patients have negative serum galactomannan tests as have anti-galactomannan antibodies, suggesting that these may bind galactomannan antigens in serum and render the galactomannan antigen test insensitive in chronic disease. In sub-acute pulmonary aspergillosis, it is unclear whether antigen or antibody tests are most sensitive, and it is probably appropriate to perform both.

Precipitation-in-gel (precipitins) techniques have been in common use for over 70 years (reviewed in [3]). These detect multiple types of immunoglobulin, including IgM in addition to IgG. They are often used with in-house antigens extracted from fungal cultures. In highly skilled hands, these may be a useful test, but the technique is time-consuming and standardization and reproduction between laboratories are extremely challenging. In the UK, this technique has a sensitivity of less than 60% and has been largely replaced by ELISA [9, 10]. In France, however, precipitins are commonly used as a “confirmatory” test in most centers [11].

In the last decade, several commercial *Aspergillus*-specific IgG ELISAs have been developed and are now widely used in CPA diagnosis. There are some important variations between these assays. Many are based on culture extract antigens similar to those traditionally used in precipitins, e.g., Immulite (Siemens, Germany), ImmunoCAP (ThermoFisher Scientific), Serion (Germany), and Genesis (UK). One assay uses a combination of unspecified recombinant antigens (Bio-Rad, USA). Another uses a combination of traditional culture extract antigens with recombinant chymotrypsin and mitogillin (Bordier, Switzerland). The ImmunoCAP and Immulite assays can only be performed on automated systems, while the other brands are available as traditional 96-well ELISA plates and can be performed manually or with the aid of automated washers.

Several recent papers describe the reproducibility of these assays. Inter-assay co-efficient of variation was 20% for the Bordier assay [12] in one study, and 3.6% for Immulite, 8.2% for Genesis, and 23–44% for Serion in another [9]. A third study describes co-efficient of variation of 5% for ImmunoCAP and 33% for Bio-Rad [10]. Inter-laboratory variation has only been described for ImmunoCAP (7.3–18.1%), which also represents batch-to-batch variation, as test kits from different manufacturing batches were used at different sites [13]. As many assays are based on culture extract antigens, establishing batch-to-batch reproducibility is essential, but to date is unavailable for other assays.

Most assays report results in arbitrary units. However, Immulite and ImmunoCAP report results in mg/L. Unfortunately, *Aspergillus* levels are consistently around twice as high by ImmunoCAP assay as by Immulite [9, 13]. It is therefore crucial that laboratories state the assay used to avoid confusion.

There is some variation in diagnostic performance between the commercial assays. In one study, the sensitivity for ImmunoCAP and Bio-Rad assays for CPA were 86 and 85%, respectively [10]. Another study compared Bordier, Bio-Rad, and Serion and found statistically significant differences in test performance with sensitivities of 97, 92, and 86%, respectively [12]. Specificities in relation to patients with chronic lung diseases were 90.3, 91.3, and 81.5% with Bordier and Bio-Rad both demonstrating statistically significantly superior performance to Serion. A third study found that ImmunoCAP and Immulite had statistically significantly superior performance to Serion, which was itself significantly superior to Genesis and Dynamiker assays [9]. Sensitivities for CPA were 96, 96, 90, 75, and 77%, respectively. Specificities in relation to healthy controls were 98, 98, 98, 97, and 99% at optimal cut-offs. Over 99% of patients in this study were positive to at least one assay, suggesting that a second assay should be performed if the first is negative and there is high degree of clinical suspicion.

The identification of optimal cut-offs is crucial for *Aspergillus* ELISA assays. In most infections, exposure to the causative organism is an uncommon event, and the presence of any antibody response represents evidence of current or past infection. However, humans are exposed to *Aspergillus* throughout life but rarely develop illness if they have structurally normal lungs and an intact innate immune system. Antibody levels increase in healthy persons throughout childhood [14], and most healthy adults have some degree of antibody response [9]. Determining a cut-off to differentiate a pathological rise in antibodies is therefore of critical importance.

Most assay manufacturers recommend a diagnostic cut-off. These cut-offs are often calculated in relation to small mixed populations with various forms of aspergillosis. As antibody levels can be much higher in chronic disease than in allergic or acute invasive disease, these cut-offs may not be optimized for CPA. A recent study identified optimal cut-offs of 1.5 AU/ml for the Bio-Rad assay, 25 mg/L for Immulite, 50 mg/L for ImmunoCAP, and 50 U/ml for Serion by performing receiver-operating curve analysis comparing 241 CPA cases to healthy European controls. For Immulite, ImmunoCAP, and Serion, these were similar to the manufacturer’s recommended cut-offs, but for Bio-Rad, this was significantly different to the recommended cut-offs of 10 AU/ml for intermediate and 15 AU/ml for positive.

CPA is found almost exclusively in patients with underlying chronic lung disease. One recent study suggests that cut-offs calculated for the Immulite assay in relation to healthy controls and controls with treated tuberculosis are similar [15]. Another study compares ImmunoCAP levels in patients with proven CPA, to those with similar clinical and radiological presentation, but with normal inflammatory markers, and identified the same cut-off (50 mg/L) as that identified elsewhere in relation to healthy controls [16]. This may not be the case for other underlying lung conditions. Median *Aspergillus*-specific IgG levels were 6 mg/L in healthy controls, but 17.4 mg/L in COPD without bronchiectasis and 35.4 mg/L in COPD with bronchiectasis [17]. To date, no studies have assessed optimal cut-offs for *Aspergillus*-specific IgG in underlying conditions other than tuberculosis, which only accounts for 15% of CPA cases in Europe [18]. Such studies are essential if these assays are to be used with confidence in CPA diagnosis.

Most CPA globally is estimated to occur secondary to tuberculosis [19] and is therefore likely to occur in resource poor settings. At present, access to *Aspergillus* serology is extremely limited in these settings [20]. Complex automated systems such as ImmunoCAP and Immulite are not ideal for resource-poor settings due to the need for maintenance. Plate ELISAs such as Bio-Rad and Bordier could be delivered at reference laboratories but are unsuitable for local clinics where most tuberculosis care is delivered, but basic facilities such as running water and electricity may be unavailable. Lateral flow device point-of-care tests have transformed the diagnosis of other conditions such as malaria and HIV in these settings. Similar assays are being developed for *Aspergillus* IgG, including assays by IMMY (Norman, OK, USA) and LDBio (Lyon, France), but to date have no data have been published on clinical accuracy and no assay has been commercialized.

In addition to diagnosis, *Aspergillus* IgG may have a role in monitoring treatment response in CPA. *Aspergillus*-IgG levels fell in seven out of ten patients treated with 6-month posaconazole in the UK and two of four treated in China [21, 22]. However, another study found that less than 10% of 25 patients treated with 6 months azole therapy in the UK had significant falls in antibody levels [10]. It is unclear whether decrease in antibody levels correlates with clinical and radiological response to treatment. More clinical validation work is needed in this area before *Aspergillus*-IgG can be considered a reliable measure of treatment response.

Given the large number of antigens present in culture extracts, it is essential to determine whether cross-reactivity occurs between *Aspergillus*-specific IgG assays and other fungi. In allergic aspergillosis, *Aspergillus* antigens have been shown to cross-react with IgE to *Penicillium, Alternaria, Cladosporium*, and *Malassezia* [23]. None of these fungi cause CPA-like disease, but it is plausible that they might generate an IgG response due to airway colonization or nail infection, resulting in false positive *Aspergillus*-specific IgG results. It is not known whether the recombinant antigens used in Bio-Rad or Bordier assays cross-react with other species. It is not known to what extent *Aspergillus*-specific IgG assays cross-react with other fungi that can cause similar clinical presentation, such as *Histoplasma, Coccidioides, and Paracoccidioides*. More work is needed in this area before *Aspergillus*-specific IgG can be used with confidence, especially in areas where other fungal lung diseases are endemic.

Most CPA in Europe is caused by *Aspergillus fumigatus* [3]*,* but in Korea, *Aspergillus niger* is responsible for 20% of cases [7]. The species diversity of CPA has not been well-described for other locations. Precipitin tests based on *A. fumigatus* are only positive in around half of aspergillosis cases caused by *A. flavus* or *A. niger* [24]. The cross-reactivity of modern commercial ELISA assays with CPA caused by other species has not been established. These issues need to be resolved before *Aspergillus* serology can be used reliably on a global basis.

### Aspergillosis in the Setting of Tuberculosis and HIV

Recent studies in Uganda suggest that chronic pulmonary aspergillosis and *Aspergillus* sensitization might be responsible for significant mortality in patients treated for tuberculosis in Uganda [10]. In an HIV setting, paired serum aliquots from 101 HIV-TB co-infected patients at the beginning and week 24 of tuberculous treatment were tested [25]. Samples were measured for *Aspergillus*-specific immunoglobulin G (IgG) and immunoglobulin E (IgE) using ImmunoCAP®; and for *Aspergillus*-specific IgG and total serum IgE using Immulite® immunoassays. The results of antibody titers between baseline and week 24 were interpreted against selected baseline characteristics. Ten percent of patients had elevated *Aspergillus*-specific IgE (indicating *Aspergillus* sensitization) and *Aspergillus*-specific IgG antibodies were elevated in 9% of the patients at the end of treatment for tuberculosis. There was a significant fall in the *Aspergillus*-specific IgG titer between baseline and week 24. Patients with a CD4 T cell count < 100 cells/μl and those who were not on anti-retroviral therapy at baseline had elevated *Aspergillus*-specific IgG antibodies (*P* = 0.01, *P* = 0.03). The ImmunoCAP® Aspergillus-specific IgG antibody titers were higher at week 24 than baseline with more positives at week 24. Pulmonary infiltrates were the commonest X-ray abnormality, and only 5% of the patients had pulmonary cavities on chest X-ray at week 24. The authors concluded that *Aspergillus* infection may complicate active pulmonary tuberculosis in HIV-positive patients and that their findings provide evidence that serology may be useful in measuring the prevalence of pulmonary aspergillosis complicating tuberculosis.

### Allergic Bronchopulmonary Aspergillosis (ABPA)

Allergic bronchopulmonary aspergillosis (ABPA) is characterized by an allergic inflammatory response to airways colonization by *Aspergillus* [26], and occasionally other fungi (see below). It is estimated that ABPA complicates around 7–14% of cases of asthma [26]. ABPA should be ruled out in every case of uncontrolled asthma. The diagnostic criteria for ABPA include the presence of bronchial asthma, immediate skin test reactivity to *A. fumigatus*, elevated total and *A. fumigatus*-specific IgE titers, pulmonary infiltrates (transient or fixed), central bronchiectasis, and peripheral blood eosinophilia [25].

A number of studies have compared different serological techniques by analyzing sera from both chronic pulmonary aspergillosis and allergic disease caused by *Aspergillus*. Baxter and colleagues assessed the performance of two commercial EIAs compared with counterimmunoelectrophoresis (CIE) [10]. In a prospective cohort study of 175 adult patients with chronic or allergic pulmonary aspergillosis, Aspergillus IgG antibodies were detected using CIE, Phadia ImmunoCap Aspergillus IgG, and Bio-Rad Platelia Aspergillus IgG. Inter-assay reproducibility was determined, and 25 patients had two serum samples analyzed within a 6-month interval. When compared with CIE, both ImmunoCap and Platelia Aspergillus IgG had good sensitivity for detection of *Aspergillus* IgG antibodies. The level of agreement between the two EIAs for positive results was good, but the concentration of antibodies was not correlated between the tests or with CIE titer. The direction of CIE titer change over 6 months was mirrored by ImmunoCap IgG levels in 92% of patients and by Platelia IgG in 72% of patients. The authors concluded that both ImmunoCap and Platelia Aspergillus IgG EIAs are sensitive measures of *Aspergillus* IgG antibodies compared with CIE. However, ImmunoCap appears to have better reproducibility and may be more suitable for monitoring patient disease. This supports previous studies.

The performance of a new commercial enzyme immunoassay for *Aspergillus* IgG (Bordier Affinity Products) compared with that of the Platelia *Aspergillus* IgG (Bio-Rad Laboratories, France) and Virion/Serion *Aspergillus fumigatus* IgG assays has recently been reported by Dumollard and colleagues [12]. This assay utilizes two recombinant antigens that share features with traditional somatic and metabolic antigens of *A. fumigatus*. The study showed that the use of recombinant, somatic, and metabolic antigens in a single EIA improved the balance of sensitivity and specificity, resulting in an assay highly suitable for use in the diagnosis of chronic and allergic aspergillosis. Notwithstanding the promising development of new serological tests, the diagnosis of allergic and chronic forms of pulmonary aspergillosis currently relies on well-established assays for *Aspergillus* IgG and IgE, in conjunction with clinical parameters.

### Allergic Bronchopulmonary Mycosis

Allergic bronchopulmonary mycosis (ABPM) is a clinical syndrome associated with immune sensitivity to various environmental fungi that occurs globally where the upper airways of asthmatic patients become colonized. A large case series has been reviewed [27]. Up to 2014 143 cases of ABPM due to fungi other than the aspergilli had been reported. The Indian sub-continent accounted for about 47% of the reported cases of ABPM, attributed predominantly to *C. albicans*, followed by Japan (16%) where *S. commune* predominates, and the remaining one-third from the USA, Australia, and Europe. The commonest etiological agent was *Candida albicans*, reported in 60% of cases, followed by *Bipolaris* species (13%), *Schizophyllum commune* (11%), *Curvularia* species (8%), *Pseudallescheria boydii* species complex (3%), and rarely, *Alternaria alternata*, *Fusarium vasinfectum*, *Penicillium* species, *Cladosporium cladosporioides*, *Stemphylium languinosum*, *Rhizopus oryzae/arrhizus*, *C. glabrata*, *Saccharomyces cerevisiae*, and *Trichosporon beigelii*. Of interest, bronchial asthma was present in only 32% of ABPM cases, whereas its association with development of ABPA is known to be much more frequent. The cases reviewed revealed a median IgE value threefold higher than that of ABPA, suggesting that the etiologic agents of ABPM evoke a stronger immunological response than that by aspergilli in ABPA [27]. ABPM is currently underdiagnosed, warranting comprehensive basic and clinical studies in order to understand its epidemiology and to develop effective therapies. The serological response to a number of the molds associated with ABPM can be monitored by ImmunoCap [27].

Demonstration of IgE and/or IgG antibodies specific for environmental molds is an important requirement for the diagnosis of ABPM (reviewed in [27]). Commercially available antigen kits for ELISA are available though not for all fungi incriminated in ABPM. There are a number of clinical and immunologic criteria for the diagnosis of allergic bronchopulmonary aspergillosis including positive specific IgE; the same may also be found in a case of simple mold-sensitization, and thus, it does not pinpoint the diagnosis of ABPM (reviewed in [27]). However, the elevation of specific IgE to a level twice as high as that in pooled sera of patients with fungal sensitization indicates ABPM (reviewed in [27]).

### Serological Differentiation Between ABPA and Allergic Bronchopulmonary Mycosis (ABPM)

Fukutomi and colleagues have addressed the issue that the presence of specific IgE to *A. fumigatus* does not always indicate genuine sensitization to *A. fumigatus* because of cross-reactivity between crude extracts (native antigen mixes) from different fungal sources [23]. The authors found that the specificity of testing could be greatly improved by measuring IgE to Asp f 1 and f 2 and characterized specific allergen components that detect genuine *A. fumigatus* allergy. It is also recognized that the problem of cross-reactivity between crude fungal extracts is also true for the identification of genuine causal fungi in individual ABPM patients. Some patients with ABPM induced by fungi other than *Aspergillus* may be consistent with ABPA diagnostic criteria because current criteria depend on IgE/IgG reactivity to crude extracts. Accurate identification of genuine causal fungi for ABPM is of clinical importance, considering that clinical presentation, anti-fungal treatment strategies, and disease prognosis can be influenced by different causal fungi. The diagnosis of causal fungi can be robustly validated by the confirmation of genuine sensitization to fungi after measuring IgE to specific allergen components, as well as repeated microbiological isolation of the fungi from their airways.

### Lateral Flow Devices for *Aspergillus* Antibody

Point-of-care (POC) testing has become the most noteworthy method of diagnosis in clinical diagnostics. Compared to centralized laboratories, POC devices provide prompt results in shorter times. Lateral flow assay (LFA)-based POC devices (immune-chromatographic technologies) are among very rapidly growing strategies for qualitative and quantitative analysis in infectious diseases. The LFA is performed over a strip, different parts of which are assembled on a plastic backing or cassette. These parts are sample application pad, conjugate pad, nitrocellulose membrane, or similar and adsorption pad. Nitrocellulose membrane is further divided into test and control lines. Pre-immobilized reagents at different parts of the strip become active upon flow of liquid sample, usually serum or respiratory secretions. LFA-based strips have different detection formats. Rapidity and one-step analysis, low operational cost, simple instrumentation, user-friendly format, less or no interferences due to chromatographic separation, high specificity, better sensitivity, long-term stability under different sets of environmental conditions, and portability of the device are unique advantages related to LFA strips. For a detail review on lateral-flow devices (LFDs), see reference [28].

Examples in the fungal world include LFDs for biomarkers: a dip stick for cryptococcal antigen and a lateral flow device for Aspergillus galactomannan-like antigen. LFDs for other fungal infections are under development.

There are numerous advantages associated with LFDs including ease of device preparation, low cost, stability over a wide range of environmental conditions, and very long shelf life; requirement of small sample volume allows sample application without pretreatment, less time of analysis, wide range of applications, and no or very little energy consumption.

However, there are a number of pitfalls including mostly qualitative or semi-quantitative, reproducibility varies from lot to lot, sometimes, and pre-treatment of sample is required which is time-consuming.

**Fig. 1:**
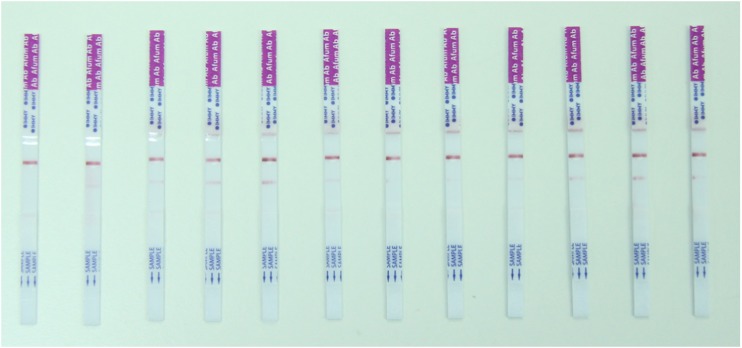
IMMY prototype LFDs for *Aspergillus* immunoglobulin using unspecified capture antigens in precipitin-positive sera from patients with chronic pulmonary aspergillosis and from precipitin-negative patients (controls). The upper band is the device control band, and the lower band is the test band

A number of LFDs for *Aspergillus* immunoglobulins are being developed, for example IMMY (Norman, OK, USA) and LDBio Diagnostics, France (Fig. 1).

### Detection of Aspergillus Antibody by Western Blotting

Oliva and colleagues have evaluated the performance of the *Aspergillus* Western blot (*Asp*-WB) IgG kit (LDBio Diagnostics, Lyon, France), a recently commercialized immunoblot assay for the diagnosis of various clinical manifestations of aspergillosis [29]. Three hundred and eight serum samples from 158 patients were tested. Specifically, 267 serum samples were from patients with *Aspergillus* disease, including 89 cases of chronic pulmonary aspergillosis, 10 aspergilloma, and 32 allergic bronchopulmonary aspergillosis, while 41 samples were from patients with *Aspergillus* colonization, including 15 cystic fibrosis (CF) and 12 non-CF patients. For blood donor controls, the *Asp*-WB specificity was 94%, while the kit displayed a sensitivity of 88.6%. Among the patients, the sensitivities of the *Asp*-WB in the diagnosis of *Aspergillus* colonization were 100 and 41.7% in CF and non-CF patients, respectively. In conclusion, the *Asp*-WB kit appeared to perform well for the diagnosis of various clinical presentations of aspergillosis in nonimmunocompromised patients.

Raised *Aspergillus*-specific IgG antibody levels play a critical role in the diagnosis of CPA. Recently developed commercial ELISAs provide a promising alternative method that can be used for the serologic diagnosis of CPA. It has been reported that the sensitivity and specificity of commercial ELISAs range from 75 to 98 and 84 to 99%, respectively.

## Does Antibody Detection Have a Role in Invasive Aspergillosis?

Pulmonary invasive aspergillosis (IPA) is a significant clinical problem in patients undergoing allogeneic hematopoietic stem cell transplantation (allo-HSCT). The measurement of cell wall components of *Aspergillus* species, such as galactomannan, is a useful diagnostic tool (reviewed in [1]). The evidence to support the use of *Aspergillus* serology in diagnosing invasive aspergillosis is less convincing.

A positive correlation of serum immunoglobulin responses against purified recombinant *Aspergillus fumigatus* proteins before allo-HSCT with the incidence of IA after allo-HSCT has been reported. In an attempt to characterize the serological response to *A. fumigatus* antigens, mycelial proteins were separated by two-dimensional gel electrophoresis [30]. The gels were immunoblotted with sera from patients with probable and proven IPA and control patients without IPA. Forty-nine different fungal proteins, which gave a positive IgG antibody signal, were identified. Furthermore, the analysis identified 18 novel protein antigens from *A. fumigatus*. These could potentially be used in the development of serological assays for invasive aspergillosis.

The clinical utility of serology for diagnosing IPA has been explored in a prospective study using a commercially available assay for the detection of anti-*Aspergillus* immunoglobulin-G (Platelia™ *Aspergillus* IgG) that has previously demonstrated high sensitivity and specificity in other patient groups [31]. In a cohort of 104 allo-HSCT recipients, anti-*Aspergillus*-IgG and *Aspergillus* antigen serum levels before allo-HSCT were measured, and weekly during their hospital stay. Overall prevalence of possible, probable, and proven IPA during hospital stay was 10, 6, and 0%. No correlation between anti-*Aspergillus* IgG levels before allo-HSCT, or after allo-HSCT, and the prevalence of IPA during hospital stay was found. Furthermore, median anti-*Aspergillu*s-IgG levels did not differ between patients with history of probable or proven IPA, as compared to patients without history of IPA. The authors concluded that the data does not support measuring anti-*Aspergillus* IgG levels for diagnosis or prediction of IPA in patients undergoing allo-HSCT.

It is possible that IgM antibodies against *Aspergillus* might have more clinical utility for the diagnosis of IPA than currently realized. A number of commercial kits for the detection of this immunoglobulin are available. A recent study evaluated the clinical value of the Dynamiker *Aspergillus*-specific IgM antibody assay in diagnosis of IPA in a multicenter prospective study that was performed in 12 hospitals in Zhejiang Province, China [32]. This assay uses galactomannan as the antibody capture molecule. A total of 59 patients were enrolled, including 30 IPA and 29 non-IPA patients. The area under the curve of the IgM assay revealed by the receiver-operating characteristic (ROC) analysis was 0.511 in the IPA cases. The study conclusion was that the *A. fumigatus*-specific IgM antibody assay was of limited value in the diagnosis of IPA.

## Allergy and Disease Caused by Filamentous Basidiomycetes

Even though an increase in infections caused by these common environmental fungi has been reported widely, the potential of common filamentous basidiomycetes as pathogens has not been explored. Basidiomycetes have recently emerged as increasingly important pathogens that incite a wide array of clinical disease manifestations including invasive as well as non-invasive diseases [33]. The authors reviewed 218 reported global cases. The most common etiologic agent was *Schizophyllum commune* in 52.3% (114/218) of the cases followed by *Hormographiella aspergillata* (*n* = 13; 5.9%), *Ceriporia lacerata* (*n* = 11; 5%), and, rarely, *Volvariella volvacea*, *Inonotus tropicalis*, *Irpex lacteus*, *Phellinus undulates*, *Perenniporia* species, *Bjerkandera adusta*, *Sporotrichum pruinosum*, *Phanerochaete steroids*, and *Cyclomyces tabacinus*. These fungi are present in the environment as gilled mushrooms, shelf fungi, and bracket fungi. The respiratory tract was the most commonly afflicted site (*n* = 71), with the majority of the cases (42; 59.1%) being allergic in etiology and comprising 34 cases of allergic bronchopulmonary mycosis. Also, *B. adusta* has been implicated in a recently described clinical entity, that is, fungus associated chronic cough, reported almost exclusively from Japan. Basidiomycete-incited diseases are currently under-diagnosed due to lack of awareness and expertise, warranting comprehensive epidemiological and susceptibility studies to determine their prevalence and to predict appropriate therapy.

Serology has been used as an adjunct test for diagnosing *S. commune* infection [34]. In patients with allergic bronchopulmonary mycosis and fungal ball, immunodiffusion detected precipitins against culture filtrate antigens prepared from the patients *S. commune* isolates. The patients’ serum was negative when antigens of *A. fumigatus*, *A. flavus*, *A. terreus*, and *A. niger* were used.

## *Talaromyces marneffei* Infection

*Talaromyces* (*Penicillium*) *marneffei* is a dimorphic fungus endemic in Southeast Asia. It can cause fatal talaromycosis (penicilliosis) in humans, particularly in HIV-infected individuals. Diagnosis of this infection is difficult because its clinical manifestations are not distinctive or specific. Specialized laboratory tests are necessary to establish a definitive diagnosis for successful management. Various several tests have been described for the detection of antibodies to *Talaromyces* (*Penicillium) marneffei*, including immunodiffusion, indirect immunofluorescence, and ELISA, but they are not widely available. These methods are specific, but less sensitive than culture.

Recent developments include the use of a cell wall mannoprotein of *Talaromyces marneffei* derived from the yeast *Pichia pastoris* expression system, designated as Mp1p in immunoassays as a capture antigen [35]. The authors report an anti-Mp1p IgG antibody ELISA with an evaluated sensitivity of 30% and a specificity of 98.5%. Furthermore, combining the results of Mp1p antigen and antibody detection improved the sensitivity of *Talaromyces* diagnosis to 100%. It was concluded that simultaneous detection of antigen and antibody using the immunoassays based on Mp1p greatly improves sensitivity and that these procedures should be useful for the routine diagnosis of *Talaromyces* infection.

## Mucormycosis

Mucormycosis is a term that encompasses infections caused by molds belonging to the order Mucorales. Traditionally, this order was assigned to the phylum Zygomycota together with the order Entomophthorales, and the different forms of disease caused by the two groups of organisms were often referred to as zygomycosis. However, following molecular analysis, the phylum Zygomycota is no longer accepted due to its polyphyletic nature. The subphylum Mucoromycotina has been proposed to accommodate the Mucorales, and the subphylum Entomophthoromycotina has been created for the Entomophthorales. Fungi of the order Mucorales can cause rhinocerebral, pulmonary, gastrointestinal, cutaneous, or disseminated disease in predisposed individuals, the different clinical forms often being associated with particular underlying disorders. Mucormycosis is the second most frequent mold infection seen in immunocompromised individuals.

There are no routine serological tests for diagnosis of mucormycosis available at present. However, a number of allergens have been described which may have potential as antigens in developing a new serological tests [36].

## Pythiosis

Oomycetes form a distinct phylogenetic lineage of fungus-like eukaryotic microorganisms. They are filamentous and microscopic. A representative example is *Pythium insidiosum*. This is pathogenic in mammals. The infection occurs mainly in tropical and subtropical areas, particularly in horses, dogs, and humans. Human infections are primarily found in Thailand (reviewed in [37]). The infection is acquired through small wounds or trauma via contact with water that contains motile zoospores or other infectious propagules (zoospores or hyphal fragments). The disease has been emerging since it was described in 1884. Depending on the site of entry, infection can lead to different forms of pythiosis, that is a cutaneous, vascular, ocular, gastrointestinal, and a rare systemic form. The infection is not contagious; no animal-animal or animal-human transmission has been reported so far. Therapy includes radical surgery, antifungal drugs, immunotherapy, or a combination of these approaches. To prevent individuals contracting the disease in endemic areas is difficult. Avoiding stagnant waters is helpful, although the presence of *P. insidiosum* on grass and soil in enzootic areas renders this practice useless.

Early diagnosis of pythiosis is very important for successful management. Clinical expertise of a clinician with the various clinical forms of the disease in different species is crucial for an early diagnosis. A positive diagnosis for *P. insidiosum* infection can be obtained in three ways: (1) determination of the presence of the agent in direct microscopy in 10% potassium hydroxide followed by culture, (2) detection of anti-*P. insidiosum* antibodies using serological assays, and (3) detection of DNA of the infectious agent in infected tissue by PCR and sequencing (reviewed in [37]).

Serodiagnosis of pythiosis can be attained by the detection of precipitating antibodies using immunodiffusion. The test is very specific but unfortunately has a low sensitivity. Other tests based on detection of antibodies include ELISA. An immunochromatographic assay and Western blot assays have been developed to increase sensitivity and specificity [37]. These tests are difficult to perform in rural areas; consequently, a hemagglutination test has been developed where sheep red blood cells coated with a *P. insidiosum* extract are tested against serum samples of patients suspected of pythiosis and the presence of agglutinating antibodies is recorded (reviewed in [37]). The test was found to be simple, rapid, and reliable for serodiagnosis of vascular and cutaneous pythiosis.

## Discussion/Conclusion

The focus of this review has been aspergillosis. Numerous *Aspergillus* antibody detection systems have been developed over the past 50 years including immunodiffusion, immunoelectrophoresis, indirect hemagglutination, and complement fixation; most commercially available assays are ELISA-based. Due to the high seroprevalence rate, an abnormal ELISA result for anti-*Aspergillus* antibodies is defined as antibody levels above those typically found in healthy individuals. Although antibody detection may be used as a supplemental test, the diagnosis of CPA and ABPA, detection of anti-*Aspergillus* antibodies in cases of acute invasive aspergillosis is limited due to profound immunosuppression and weakened humoral response in these high-risk patients. The sensitivity and performance of *Aspergillus* IgG antibody testing for diagnosis of aspergillosis are summarized here. The best IgG assays have a 90–95% sensitivity for chronic pulmonary aspergillosis and aspergilloma caused by *A. fumigatus*, much more sensitive than culture, even where high-volume culture techniques are used. The antibody titer varies widely: 30–50% of patients with ABPA have detectable *A. fumigatus* antibodies, usually at low titer. High titers suggest the complication of chronic pulmonary aspergillosis, which needs to be correlated with radiology. Monitoring of treatment response in patients with *Aspergillus* disease by serology is useful. Falling IgG titers (for example, by ImmunoCap) is useful as a measure of therapeutic response in chronic pulmonary aspergillosis and *Aspergillus* rhinosinusitis. The rate of fall is slow and takes weeks or months even in patients doing well. Lack of fall is suspicious of treatment failure.

Fungal rhinosinusitis is more often caused by *A. flavus* than *A. fumigatus*. Detection of *A. flavus* IgG antibody is a useful confirmatory evidence of infection, especially if cultures are negative but histology or direct microscopy positive. Patients with *Aspergillus* bronchitis often have positive IgG antibodies (and negative IgE antibodies), but *A. fumigatus* is slightly less common as the cause, so non-*A. fumigatus* IgG antibodies (precipitins) should be monitored if antigens are available.

IgE antibody testing against *A. fumigatus* is useful to detect *Aspergillus* sensitization. The skin prick test against *A. fumigatus* is more sensitive than blood testing. Either is required for the diagnosis of ABPA (usually both are positive) and are usually positive at a much lower level in patients with severe asthma with fungal sensitization (SAFS). This test could be a useful screening test in asthmatic patients to detect ABPA or SAFS, if skin testing is not performed. Some patients with other *Aspergillus* diseases, notably chronic pulmonary aspergillosis, have positive *Aspergillus* IgE antibody titers. Occasionally, this test is positive when the IgG antibody test is negative, which is helpful.

There are numerous marketed IgG antibody tests to detect *A. fumigatus* antibodies, including Immy, Serion/Virion, Bioenche, Bio-Rad, Thermofisher, Elitech, Microgen, Meridian LD Bio, and Siemens. A number of these have been evaluated by the authors and summarized above.

The sensitivity of anti-*Aspergillus* immunoglobulin (Ig)G ELISAs in severely neutropenic individuals with proven or probable IPA ranges from 6 to 84% depending on the target antigen (e.g., cell lysate, purified, or recombinant antigens) used and the patient population tested. The inconsistency of these data suggests that detection of antibodies to *Aspergillus* alone cannot be relied on as an accurate diagnostic marker for acute IPA in this patient population.

In summary, detection of elevated anti-Aspergillus antibodies can be used as a diagnostic marker for noninvasive disease in immunocompetent or non-neutropenic individuals. However, these assays have a limited clinical utility in immunosuppressed patients, for whom additional laboratory evidence (e.g., fungal culture, radiographic signs) is necessary. Notably, detection of this analyte is absent from recently developed algorithms for differentiating Aspergillus colonization from invasive disease in intensive care patients.

Basidiomycetes appear to be increasingly implicated in allergic and chronic fungal disease as well, being reported as causing systemic infections in vulnerable patients. Accumulating evidence suggests that serological tests may be useful in the composite phenotyping of these patients. The characterization of specific antigens of *Talaromyces* and their use in formulating serological tests is an important advance in the diagnosis of this infection. The agents of mucormycosis, a potentially life-threatening infection, are not detected by the widely used fungal biomarkers such as galactomannan or β-1-3-d-glucan. The key to a favorable clinical outcome lies in the development of laboratory tests that enable early diagnosis and timely treatment of mucormycosis. Several studies have looked at antigen-based and PCR-based tests for Mucorales. Despite many years of research, there are no major advances in the serological diagnosis of mucormycosis. Recent developments in the diagnosis of pythiosis in human subjects include a hemagglutination assay that has been modeled on an accumulating understanding of humoral responses to *Pythium*.

Point-of-care (POC) testing is an exciting development, especially for developing countries with limited laboratory resources. Compared to centralized labs, POC provides prompt results in shorter times. Lateral flow assay (LFA)-based POC devices are among very rapidly growing strategies for qualitative and quantitative analysis. The excellent features and versatility of these strips and cassettes make them an ideal choice for point-of-care applications. Lateral flow assay basically combines a number of variants such as formats, biorecognition molecules, labels, detection systems, and applications.

No doubt, LFA strips have a broad range of applications in clinical mycology. Most LFAs give qualitative or semi-quantitative results which can be observed by the naked eye. Conventional LFAs are normally qualitative and give answers yes or no. A good LFA biosensor should have a number of attributes such as biocompatibility, high specificity, high sensitivity, rapidity of analysis, reproducibility/precision of results, wide working range of analysis, accuracy of analysis, high throughput, compactness, low cost, simplicity of operation, portability, flexibility in configuration, possibility of miniaturization, and potential of mass production and on-site detection. We await further progress in the development of these devices for detecting fungal antibodies.
